# Results of screening in early and advanced thoracic malignancies in the EORTC pan-European SPECTAlung platform

**DOI:** 10.1038/s41598-022-12056-0

**Published:** 2022-05-18

**Authors:** M. Morfouace, S. Novello, A. Stevovic, C. Dooms, U. Janžič, T. Berghmans, R. Dziadziuszko, T. Gorlia, E. Felip, L. Paz-Ares, J. Mazieres, M. O’Brien, P. Bironzo, J. Vansteenkiste, L. Lacroix, A. C. Dingemans, V. Golfinopoulos, B. Besse

**Affiliations:** 1 EORTC HQ, Avenue E. Mounier 83/11, 1200 Brussels, Belgium; 2 Department of Oncology, University of Turin, AOU San Luigi, Orbassano (TO), Italy; 3 Department of Respiratory Diseases and Respiratory Oncology Unit, University Hospitals KU Leuven, Leuven, Belgium; 4 Department of Medical Oncology, University Clinic of Pulmonary and Allergic Diseases Golnik, Golnik 36, Golnik, Slovenia; 5 Thoracic Oncology Clinic, Institut Jules Bordet, Université Libre de Bruxelles, Brussels, Belgium; 6 Department of Oncology and Radiotherapy, and Early Clinical Trials Unit, Medical University of Gdansk, Gdansk, Poland; 7 Oncology Department, Vall d’Hebron University Hospital and Vall d’Hebron Institute of Oncology (VHIO), Barcelona, Spain; 8 Hospital Universitario 12 De Octubre, Madrid, Spain; 9 Service de Pneumologie, CHU de Toulouse, Université Paul Sabatier, Toulouse, France; 10 Lung Unit, Royal Marsden Hospital, Imperial College, London, UK; 11 Department of Medical Biology and Pathology, BMO Unit From AMMICa UMS3655/US23, Gustave Roussy, Villejuif, France; 12 Department of Respiratory Medicine, Erasmus MC Cancer Institute, University Medical Center, Rotterdam, The Netherlands; 13 Department of Medical Oncology, Gustave Roussy, Villejuif, France

**Keywords:** Cancer screening, Non-small-cell lung cancer

## Abstract

Access to a comprehensive molecular alteration screening is patchy in Europe and quality of the molecular analysis varies. SPECTAlung was created in 2015 as a pan-European screening platform for patients with thoracic malignancies. Here we report the results of almost 4 years of prospective molecular screening of patients with thoracic malignancies, in terms of quality of the program and molecular alterations identified. Patients with thoracic malignancies at any stage of disease were recruited in SPECTAlung, from June 2015 to May 2019, in 7 different countries. Molecular tumour boards were organised monthly to discuss patients’ molecular and clinical profile and possible biomarker-driven treatments, including clinical trial options. FFPE material was collected and analysed for 576 patients with diagnosis of pleural, lung, or thymic malignancies. Ultimately, 539 patients were eligible (93.6%) and 528 patients were assessable (91.7%). The turn-around time for report generation and molecular tumour board was 214 days (median). Targetable molecular alterations were observed in almost 20% of cases, but treatment adaptation was low (3% of patients). SPECTAlung showed the feasibility of a pan-European screening platform. One fifth of the patients had a targetable molecular alteration. Some operational issues were discovered and adapted to improve efficiency.

## Introduction

Thoracic malignancies are the most common cause of cancer related death in the world. Lung cancers represent more than 11% of all cancer cases^1^, with more than 2 million new cases in 2020. Mortality linked to lung cancers reached 18% of all cancer deaths for both genders^1^, which represents around 388,000 deaths in Europe in 2018^2^. The majority of lung cancers are diagnosed at an advanced stage and the mortality in patients diagnosed with lung cancer is above 80%, including all stages and histologies. Non-small cell lung cancer (NSCLC) is the most common histological type (84%^3^) comprising mainly of the adenocarcinoma and squamous cell carcinoma subtypes. Rarer thoracic malignancies include thymic carcinoma, mesothelioma, and neuro-endocrine tumours. The discovery of oncogenic driver mutations in NSCLC has improved the treatment landscape, moving it toward biomarker-driven treatments. Driver alterations include predominantly *EGFR* and *KRAS*, but also *RET*, *BRAF*, *MET* (exon 14 skipping) mutations, *HER2* amplifications and *ALK*, *ROS1*, *RET, NRG1* and *NTRK1/2/3* fusions.

Across Europe, we observe a diversity of guidelines for biomarker testing for advanced NSCLC varying from recommended testing of a few genes and proteins (*EGFR*, *ALK*, *ROS1* and PD-L1 in England) to testing 11 genes (including *NTRK1/2/3* and *ROS1*) in France and the Netherlands^4^.

SPECTA, the European Organisation for Research and Treatment of Cancer (EORTC) translational research platform, launched a program in 2015 called SPECTAlung, to perform molecular screening for patients with thoracic malignancies. The goal was not only to better understand the molecular landscape of thoracic malignancies in Europe but also to provide access to biomarker testing for patients with no or limited access to comprehensive molecular screening technology.

Here we are reporting the results of the SPECTAlung program in terms of platform quality metrics, molecular landscape and actionability.

## Results

### SPECTAlung program quality metrics

**Figure 1:**
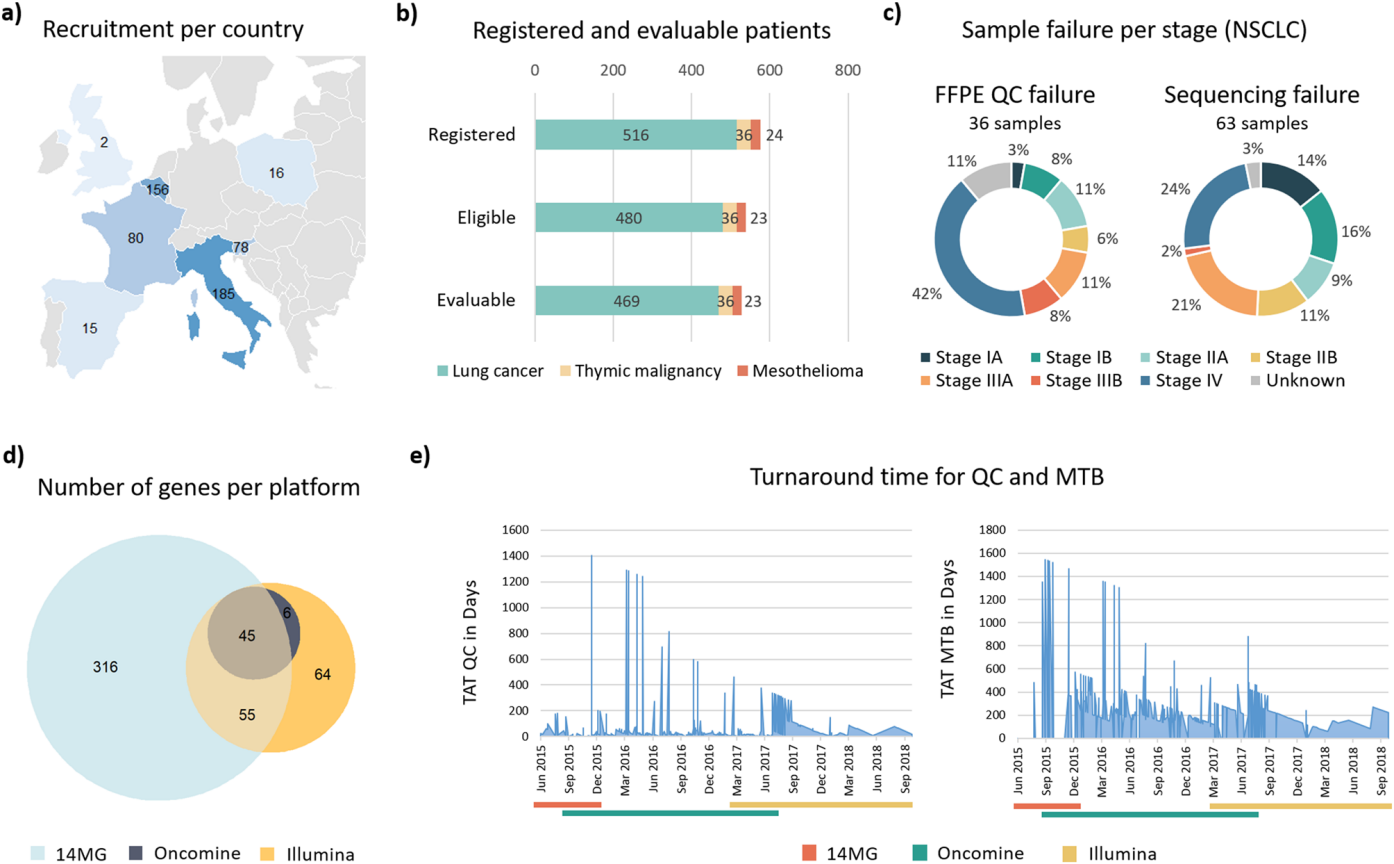
Quality metrics for the SPECTAlung platform. (**a**) Map of recruiting countries and number of patients enrolled per country (**b**) Number of registered, eligible, and evaluable patients per disease (**c**) FFPE QC (left) and sequencing (right) failure per stage in NSCLC, at the sample level. Colour code on the graph and in the legend are in the same order. (**d**) Number of genes covered by each platform, including overlapping genes. (**e**) Turnaround time between patient registration and pathology FFPE QC (left) or molecular tumor board (right). The bar below the graph represents the different platform used.

The SPECTAlung program ran for almost 4 years and recruited 576 patients. The recruitment took place in 7 countries (Italy, Belgium, France, Slovenia, Spain, Poland and UK, Fig. 1a). At the central biobank, 36 quality failures (6.2%) were recorded for lung cancer (out of 577 samples from 516 screened patients), no FFPE QC failure for thymic carcinoma (38 samples for 36 patients), and only 1 for mesothelioma (3.8%, out of 26 samples received for 24 patients). Therefore 480 patients with NSCLC, 36 patients with thymic malignancy and 23 patients with mesothelioma were eligible in SPECTAlung.

For lung cancer patients, sequencing failures were also observed: 63 samples failed sequencing in total. However, as sites could re-send new material in case of sequencing failure, only 11 overall failures were observed at the patient level. Similarly, sequencing failure was observed for 1 thymic sample (but another sample was successfully analysed for this patient) and none for mesothelioma samples. Altogether, 469 patients were evaluable in the lung cohort, 36 patients with thymic malignancies and 23 patients with pleural mesothelioma (Fig. 1b). Overall, we observed only 8.6% of failure (at the patient level). Most failures were observed for samples from patients with stage IV NSCLC (15 out of 36 of sample QC failures, 42%, Fig. 1c, left panel and 24% of all screening failures, Fig. 1c, right panel).

Clinical data at baseline (demographics, medical history, current disease status) was fully completed for 96% of patients. For 21 patients (4%), missing data on patient medical history or primary disease and biomarkers was reported. Follow-up data was completed for all patients, with a median OS follow-up time of 21.4 (19.6, 22.6) months (Sup. Figure 1). At time of database lock, 269 patients were alive, 186 dead and 84 were lost to follow-up.

Samples were successfully analysed for 31 patients using the 14MG platform, 342 patients using the Oncomine focus panel and 145 patients with the Illumina TST-170 NGS assay. Samples from six patients (5 lung, 1 mesothelioma) were successfully analysed by both 14MG and Illumina and 4 lung cancer samples with Oncomine and Illumina.

Data on 45 genes were available across all three diagnostic panels (Fig. 1d and Sup. Table 1 for gene list). Therefore, for the combined molecular analysis, we focussed on those 45 genes, with the addition of *TP53*, which was not sequenced with Oncomine focus panel.

**Table 1:** Clinical characteristics of the patient population (eligible population).

	NSCLC n = 480 (%)	TM n = 36 (%)	Mesothelioma n = 23 (%)
Sex	289 M, 189 F, 2 missing	21 M, 15 F	14 M, 9 F
Age at Dx	Median 65 (18–86)	Median 49 (27–82)	Median 63 (23–79)
Age at registration	Median 65 (19–87)	Median 51 (31–83)	Median 63 (23–80)
**Smoking history** Non-smoker Ex-smoker Current smoker Unknown	83 (17.3%) 327 (68.1%) 65 (13.5%) 5 (1%)	16 (44.4%) 15 (41.7%) 2 (5.6%) 3 (8.3%)	11 (47.8%) 11 (47.8%) 0 1 (4.3%)
**Environmental** Asbestos Chemical agent Radiation	19 (4%) 18 (3.8%) 1 (0.2%)	1 (2.8%) 0 0	12 (52.2%) 0 1 (4.3%)
Histology (WHO)—From PATH CRF	Adenocarcinoma: 300 (62.5%) Squamous cell carcinoma: 104 (21.7%) Other (incl. large cell carcinoma): 76 (15.8%)	Type AB: 3 (8.35%) Type B1: 4 (11.1%) Type B2: 7 (19.45%) Type B3: 4 (11.1%) Type C: 14 (38.9%), including 2 epidermoid non-keratinizing carcinoma, 2 basaloid carcinoma, 2 undifferentiated and 7 unknown Unknown: 4 (11.1%)	Epithelioid: 18 (78.3%) Sarcomatoid: 1 (4.3%) Biphasic: 2 (8.7%) Desmoplastic: 1 (4.3%) Unknown: 1 (4.3%)
Stage at registration (or diagnosis if registration if registration unknown)	(AJCC7) 0—1 (0.2%) IA—84 (17.5%) IB—53 (11%) IIA—71 (14.8%) IIB—46 (9.6%) IIIA—92 (19.2%) IIIB—22 (4.6%) IV—101 (21%) Unknown—10 (2.1%)	(Masoka) Stage I—6 (16.7%) Stage IIb—1 (2.8%) Stage III—8 (22.2%) Stage IVa—9 (25%) Stage IVb—9 (25%) Unknown—3 (8.3%)	Grade 1—7 (30.4%) Grade 2—1 (4.3%) Grade 3—2 (8.7%) Unknown—13 (56.5) Stage I—2 (8.7%) Stage IB—1 (4.4%) Stage II—5 (21.7%) Stage III—7 (30.4%) Stage IV—7 (30.4%) Missing—1 (4.4%)
Localization	Main Bronchus—15 (3.1%) Upper lobe—245 (51%) Middle lobe—16 (3.3%) Lower lobe—159 (33.1%) Other bronchus—14 (2.9%) Other—24 (5%) Unknown—7 (1.4%) Side: Right—253 (52.7%) Left—215 (44.8%) Both—1 (0.2%) Unknown—11 (2.3%)	Thymus—18 (50%) Mediastinum—17 (47.2%) Other—1 (2.8%) Side: Anterior—21 (58.3%) Middle—1 (2.8%) Posterior—2 (5.6%) Unknown—12 (33.3%)	Parietal—18 (78.3%) Both—4 (17.4%) Other—1 (4.3%) Side: Right—18 (78.3%) Left—5 (21.7%)
Curative surgery	Yes: 368 (76.7%) No: 110 (22.9%) Unknown: 2 (0.4%)	Yes: 23 (63.9%) No: 13 (36.1%) Unknown: 0	Yes: 6 (26.1%) No: 17 (73.9%) Unknown: 0
Curative RT	Yes: 80 (16.7) No: 398 (82.9%) Unknown: 2 (0.4%)	Yes: 20 (55.6%) No: 16 (44.4%) Unknown: 0	Yes: 4 (17.4%) No: 19 (82.6%) Unknown: 0
Chemotherapy	Yes: 233 (48.5%) No: 245 (51%) Unknown: 2 (0.4%)	Yes: 23 (63.9%) No: 13 (36.1%) Unknown: 0	Yes: 13 (56.5%) No: 10 (43.5%) Unknown: 0

The median turn-around time (TAT) between patient registration in the program and sample passing QC at the central biobank was 18 days (Fig. 1e, left panel), but with a wide range (from 1 to 1405 days). This variability was mostly linked with delay in site shipping the FFPE material to the central biobank and the change in biobank that happened in August 2017. Similarly, the TAT from patient registration to molecular tumour board (MTB) was 214 days, ranging from 56 to 1545 days (Fig. 1e, right panel). This very long TAT can also be explained by the switch in central lab (14MG, Gustave Roussy, Almac Diagnostic) and was clearly an issue to inform treatment of advanced stage patients.

### Patients characteristics

The SPECTAlung program enrolled samples form 539 individual patients, diagnosed with NSCLC (n = 480, 89%), thymic malignancies (n = 36, 6.7%) and pleural mesothelioma (n = 23, 4.3%). Patient characteristics can be found in Table 1. Overall survival (from date of cancer diagnosis) is displayed in Supplementary Fig. 1.

For NSCLC, median age at diagnosis was 65, the gender ratio of males to females was 1.5, and more than 75% were either current or former smokers, as expected for this patient population. 62.5% of NSCLCs were adenocarcinomas and 21.7% were of the squamous cell subtype. All stages were analysed within this program, and equally distributed (stage at registration: 28.5% stage I, 24.4% stage II, 23.8% stage III, 21% stage IV).

For thymic malignancies, median age at diagnosis was 49, with a 1.4 male to female ratio and around 50% of the patients were current or former smokers. Despite the small number of cases recruited in the program, we analysed samples representing most subtypes and stages.

Finally, our limited pleural mesothelioma population had a median age at diagnosis of 63, and a similar male to female ratio (1.55). Around 50% of the patients were former smokers, and 50% of the population had a known exposure to asbestos, the most important risk factor for this disease. Different subtypes of mesothelioma were analysed, from all stages.

**Figure 2:**
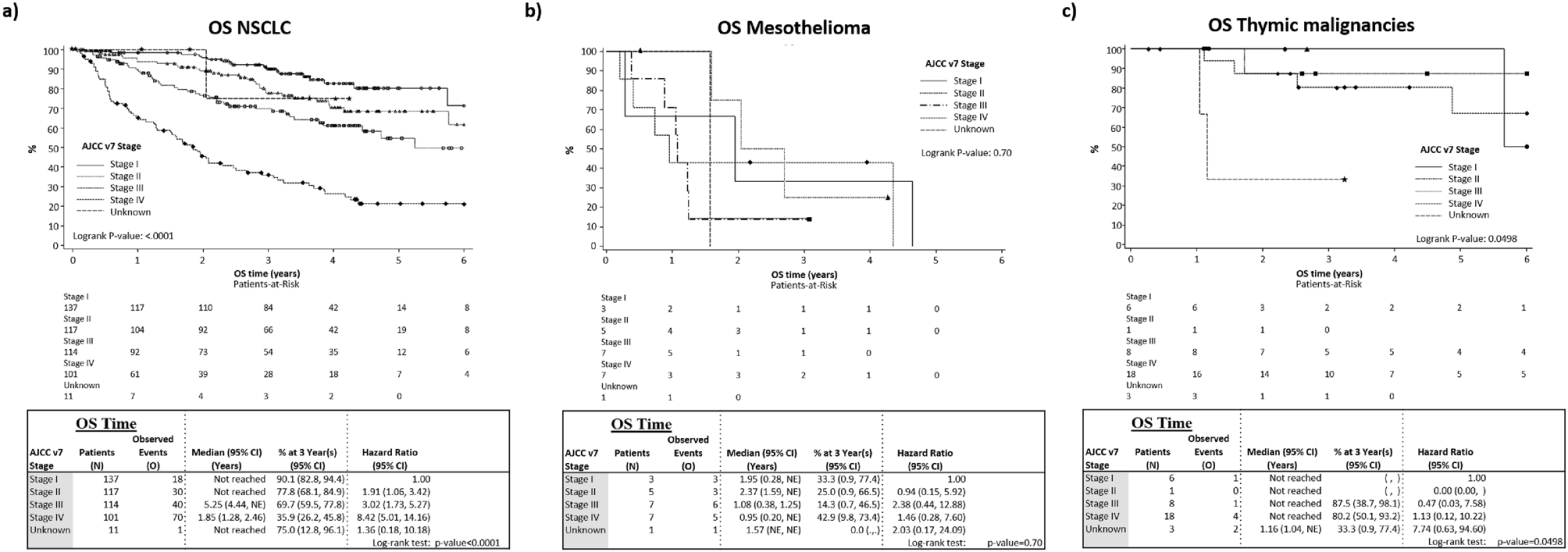
OS Kaplan Meier curve by AJCC v7 Stage. together with a summary of associated statistics (median OS, 3-year OS rate estimates including the corresponding two-sided 95% confidence interval intervals (calculated by Greenwood formula’s estimation of the standard deviation for rates and by Brookmeyer and Crowley technique for the median). OS censoring markers were displayed. Cox’s proportional hazards model was fit by AJCC v7 stage. Hazard ratios (HR) with 95% confidence intervals were computed with AJCC v.7 stage I as reference stratum (HR = 1.00). Log-rank test was computed at 5% significance. NE: Not Estimated. (**a**) NSCLC. There was a significant difference between stage (*p* < 0.0001). (**b**) Mesothelioma. There was no significant difference between stage (*p* = 0.70). (**c**) Thymic malignancies. There was a significant difference between stage (*p* = 0.0498).

Overall survival (OS, from date of cancer diagnosis) is displayed in Fig. 2. OS for NSCLC is as expected, however, due to few events in thymoma and mesothelioma populations, the curves are less informative.

### Molecular characterisation of the NSCLC, mesothelioma and thymic malignancies

**Figure 3:**
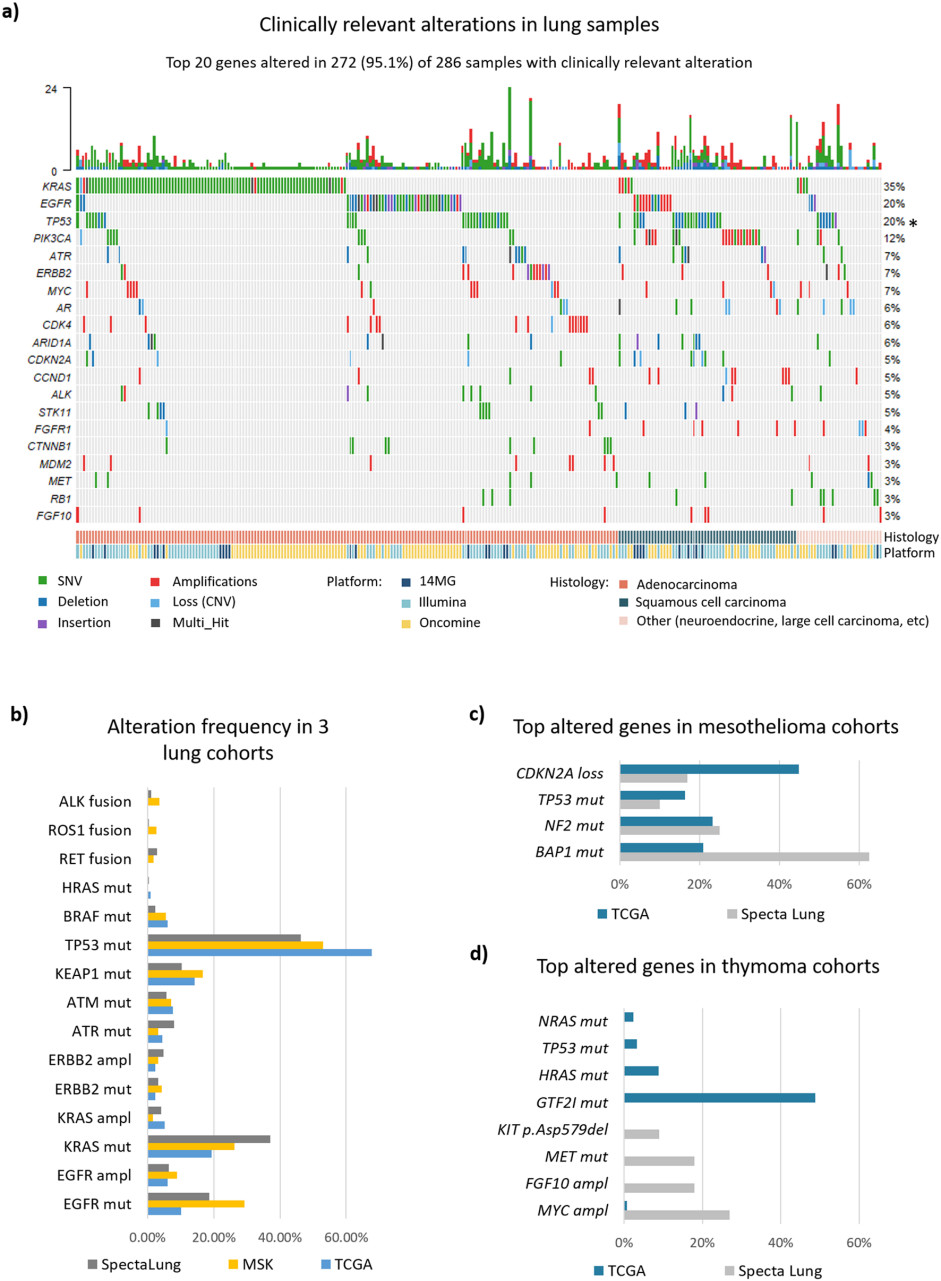
Molecular landscape of patients. Only clinically relevant molecular alterations (SNVs, indels, CNVs) are represented. (**a**) Top 20 altered genes (y axis) in NSCLC patients (sorted by histology on the x axis). The platform used to sequence each sample is also represented on the x axis. The bar plots above the graph represent the mutation rate for each sample. Alterations are color coded by type (SNV in green, deletion in dark blue, insertion in purple, amplification in red, loss in clear blue and multi-hit in brown. Note: (*) TP53 gene is not covered by Oncomine platform. (**b**) Alteration frequency for clinically actionable alterations for NSCLC patients from SPECTAlung (grey bar), MSK (yellow bar) or TCGA (blue bar) cohorts. Frequency calculations for SPECTAlung cohort are adjusted for *TP53*, *ATM* and *KEAP1* genes to include only samples covered by platforms that screened those genes (Illumina &14MG for *TP53* and *ATM*, 14MG only for *KEAP1*) (**c**) Comparison of the mutational landscape of mesothelioma samples in SPECTAlung with top altered genes in TCGA cohort (87 patients). Note: (*) TP53 gene is not covered by Oncomine platform, NF2 and BAP1 covered only by 14MG. Frequency calculations are adjusted for those genes. (**d**) Top altered genes in the SPECTAlung thymoma population and TCGA thymoma cohort (123 patients). Note: GTF2I oncogene is not covered by any panel used in this study.

Molecular analysis was successful for 480 patients, with clinically relevant molecular alterations (SNVs + INDELs + CNVs) detected in 286 samples. The top 20 altered genes (19 included in all 3 panels and *TP53*) are presented on the oncoplot (Fig. 3a). The most frequently altered genes were *KRAS* (mutated in 29% of adenocarcinoma and 1.9% of squamous cell carcinoma patients), *EGFR* (mutation in 6.7% of adenocarcinoma and 1.9% of squamous cell carcinoma but amplification in 2.3% of adenocarcinoma and 8.6% of squamous cell carcinoma patients) and *TP53* (9.3% of adenocarcinoma and 21.1% of squamous cell carcinoma). PIK3CA is amplified in 13.4% of squamous cell carcinoma only. On the contrary, CDK4 amplification is only found in 5.3% of adenocarcinoma. The mutational pattern observed in our population is independent of stage for all histologies. Comparing with two other cohorts (TCGA^5^, composed mainly of early stage disease and MSKCC^6^, composed mainly of advanced stage disease), the top alterations from SPECTAlung are found within similar range, with the exception of *TP53*, that was altered more often in the TCGA and MSKCC cohort. Numbers of targetable driver alterations are comparable within the 3 cohorts, except for the low number of fusions identified within the SPECTAlung program (Fig. 3b). No co-mutations were observed between *KRAS* and *EGFR* in this cohort.

A limited number of patients entering the SPECTAlung program had a diagnosis of mesothelioma (n = 23, 25 samples analysed) or thymic malignancies (n = 36, 37 samples analysed).

For mesothelioma, alterations were found in 12 out of 25 samples (48%). The most common alterations were *BAP1* mutations (n = 5; of note, *BAP1* was assessed only by the 14MG panel and only in the tumour specimen, no germline analysis), *CDKN2A* loss (n = 2) and *NF2* mutation (n = 2; to note, *NF2* was assessed only by the 14MG panel) (Fig. 3c). Despite the low number of mesothelioma samples in this cohort, the results are similar to the one from the TCGA cohort^7^ (Fig. 3c), where high prevalence for alterations in those 4 genes was also found.

For thymic malignancies, clinically relevant alterations were found in only 10 samples (out of 37 samples, 27%) (Fig. 3d): 3 *MYC* amplification, 2 amplifications in the cell cycle pathway (1 for *CDK4* and 1 for *CDK6*), and 1 pathogenic alteration in *KIT* (p.Asp579del). However, when comparing with the top genes altered in the TCGA cohort^7^, no overlap was seen. Our cohort is extremely limited and importantly, *GTF2I*, the most altered gene in the TCGA cohort, was not assessed in any of the panels used in this program.

### Molecular tumour board and treatment orientation

**Figure 4:**
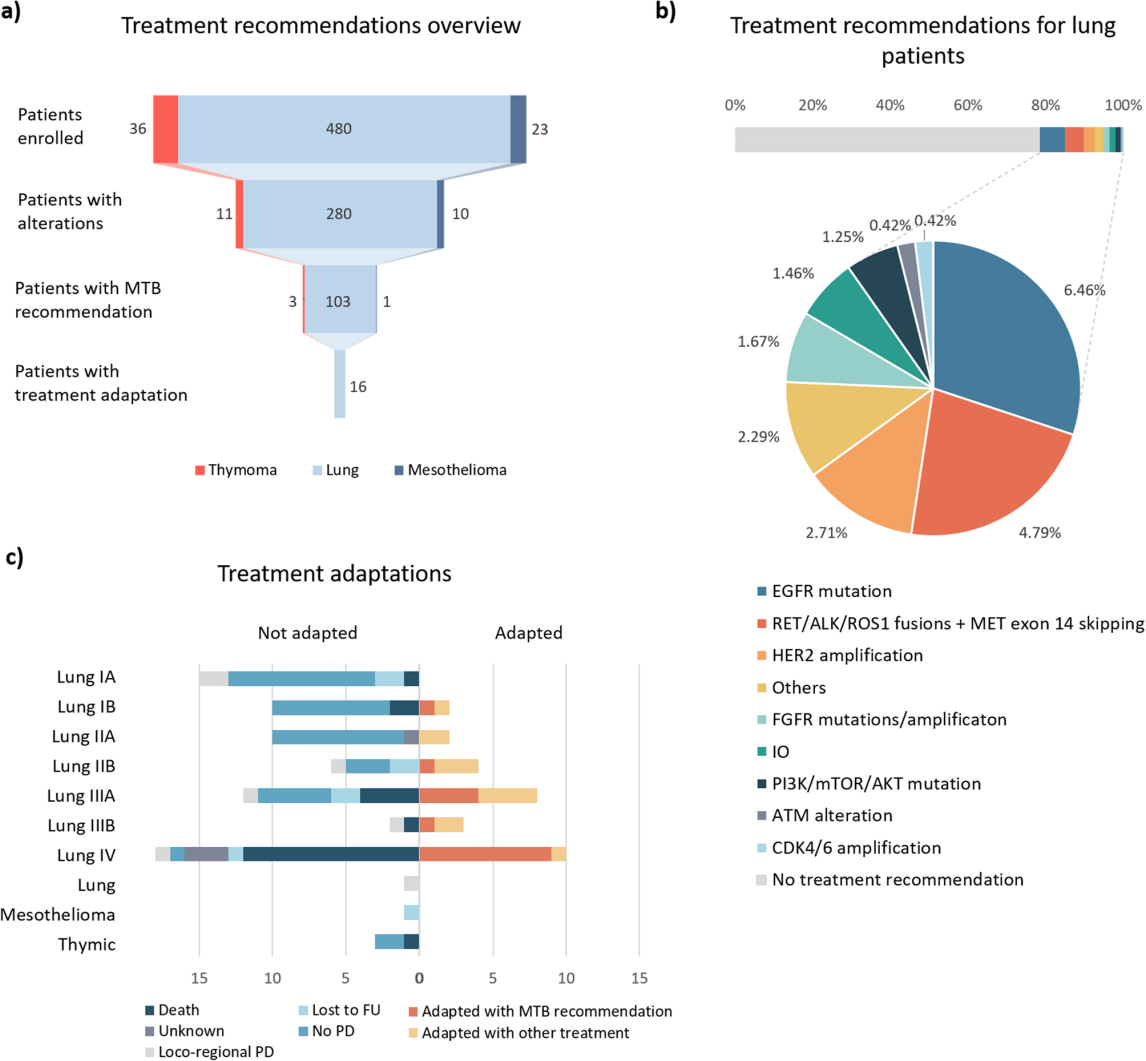
Actionability and limits of the platform. (**a**) Attrition rate between patient enrolled to treatment adaptation. Thymoma in red, NSCLC in clear blue and mesothelioma in dark blue. (**b**) Treatment recommendations for NSCLC patients. The different targetable alterations are color-coded and enlarged in the pie chart below. (**c**) Main reason for treatment adaptation (right) or absence of adaptation (left) per stage, for NSCLC patients and globally for other diseases.

Molecular tumour board meetings were organised to discuss treatment options for every patient and were composed of expert clinicians, molecular biologists and pathologists. Treatment recommendations were possible only for 107 patients (19.9%) (Fig. 4a), mainly for lung malignancies (103 patients) but also for 1 mesothelioma patient and 3 thymic malignancies.

These recommendations were based on a defined spectrum of molecular alterations that have well-known treatment options for NSCLC such as *EGFR* mutations, *HER2* amplifications, MET exon 14 skipping or *RET/ALK/ROS1* fusions (Fig. 4b). The IO recommendations (PD-(L)1 inhibitors) were based on a TMB above 16 (calculated only for Illumina analysis), following findings from the POPLAR and OAK trials.

From the 103 recommendations, adaptation of treatment was reported only for 16 patients (15.5% of cases). Reasons for not adapting treatment are displayed in Fig. 4c. The main reason for early stages (I to IIb) was that few recurrences were observed: 2 out of 15 patients with stage Ia (13.3%); 2 out of 10 patients with stage Ib (20%); none for patients with stage IIa; and 1 out of 10 patients for stage IIb (10%). While in later stages (IIIb and IV), the main reason was due to patient death: 12 out of 28 patients with stage IV (42.8%) and 1 out of 5 patients with stage IIIb (20%). For stage IIIa, both reasons applied: 4 out of 20 patients died (20%) and 5 out of 20 patients had no progression (25%).

### Molecular alterations and smoking status in the population of patients with NSCLC

**Figure 5:**
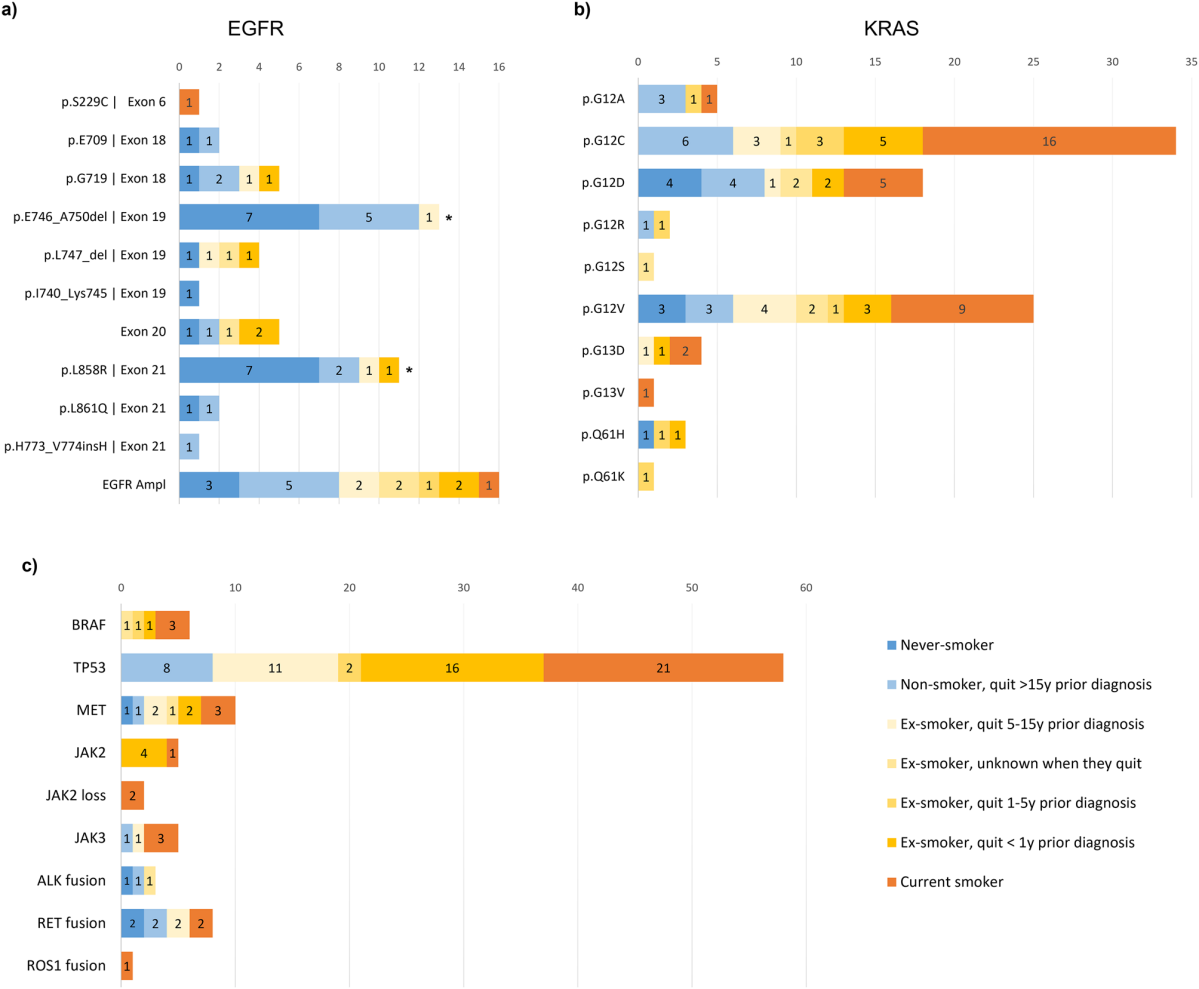
Smoking status and molecular alterations. (**a**) EGFR mutations in exon 6, 18, 19 and 21 found in the current smokers (red), former smokers (yellow) and never smokers (blue). (**b**) KRAS mutations in exon 12, 13 and 61 found in the current smokers (red), former smokers (yellow) and never smokers (blue). (**c**) BRAF, TP53, MET mutations, JAK2 mutations and loss and fusions in ALK, RET and ROS1 found in the current smokers (red), former smokers (yellow) and never smokers (blue). NOTE: For alterations with *, BH adjusted p value from Fisher’s exact test was < 0.05.

In our population, 81.6% of the patients were either former or current smokers at time of diagnosis. Our cohort confirmed the fact that most patients harbouring an *EGFR* mutation in exons 18, 19 or 21, are either never smokers (Fig. 5a, dark blue) or former smokers that stopped more than 15 years prior to diagnosis (Fig. 5a, light blue). Furthermore, statistical analysis confirmed association between non-smoking status and *EGFR* p.E746_A750del (*p* = 0.000028) and p.L858R (p = 0.0019) mutation. Only 2 active smokers with NSCLC (squamous cell histology) showed aberrations in EGFR, one with an *EGFR* amplification, the other a with a mutation in exon 6 (Fig. 5a, red). For *EGFR* exon 20, mutations were seen in both former smoker and never smoker population (Fig. 3a, orange and blue): 2 p.T790M mutation, 1 p.S768I mutation and 2 p.Ala767_Ser768insSerValAsp mutations. *EGFR* mutations were observed across all stages and no co-mutations in the *EGFR* gene were observed in this cohort.

The association between smoking status and *KRAS* mutations is different than for *EGFR*. 94 mutations were identified in *KRAS*, most of them on exon 2, either at the codon 12 position (85 out of 94), codon 13 (n = 5) or codon 61 (n = 4). The most prevalent mutations were p.G12C (n = 34), p.G12V (n = 25) and p.G12D (n = 18). The most prevalent mutations were observed in both smokers and non-smokers, with a higher prevalence in the first subgroup (Fig. 5b). As example, the p.G12C mutation, 17.6% of carriers were never smokers and 82.4% were current or former smokers, in line with the smoking profile of the global lung population of this program. The p.G12V mutation has a similar profile as the p.G12C mutation but with no specific trend toward smoking status (24% of carriers are never smokers).

For the p.G12D mutation, almost half of carriers are never smokers (44%). Looking in the TCGA cohort, this mutation was observed in 50% of non-smokers (10 out of 20) whereas the other codon 12 mutations were carried in 73% (p.G12C) and 74% (p.G12V) of smokers, in line with the global smoking prevalence of the TCGA population (28.2% of non-smoker and reformed smokers for longer than 15 years) (Supplementary Fig. 2a). Finally, a similar trend is observed in the MSKCC cohort with the p.G12D mutations found in 30% of non-smokers, whereas the p.G12C mutation was present only in 12% of non-smokers and p.G12V in 17% (Supplementary Fig. 2b).

Unlike with *EGFR* alterations, the association between other driver alterations and smoking status remains unclear (Fig. 5c). *ALK* and *RET* fusions seem to be predominantly associated with non-smokers, but only a limited number of fusions were detected in SPECTAlung (3 *ALK*, 8 *RET* and 1 *ROS1*). The *BRAF* mutations (1 p.V600E, 1 p.K601N, 1 p.G466A, 1 p.D594N, 1 p.G469R, 1 p.P299ThrfsTer) and *JAK2* alterations were all identified in smokers, whereas *TP53*, *MET* and *JAK3* mutations were identified independently of smoking habits.

## Discussion

This pan-European platform, focusing on thoracic malignancies, ran for almost 4 years (June 2015–May 2019) and recruited 576 patients from 10 clinical sites, in 7 European countries.

The attrition rate due to sample QC failure or sequencing failure was limited, with more than 80% of the registered patients evaluable. This low failure rate might be explained by the relatively high volume of surgical specimens submitted for analysis (26.4% of samples) and repartition of disease stage with less than 25% of NSCLC patients with advanced disease. The completion of the database for patient baseline characteristics and follow-up is of good quality, with a median overall survival follow-up of almost 2 years and only 15.5% of patients lost to follow-up.

The molecular landscape of NSCLC was comparable to other studies (TCGA or MSKCC). *EGFR* mutations were found in 6.5% of NSCLC patients, which is somewhat lower than the general prevalence of *EGFR* alterations in NSCLC (around 10–15% in European cohorts^8,9^). In the MSKCC study, they found 26.9% of patients with *EGFR* alterations, probably due to a higher rate of never smokers in this study or preselection of subjects. One explanation for the lower prevalence in our cohort was that patients with a high chance of an *EGFR* mutation (e.g. adenocarcinoma and never smokers) might have had a prior local testing^10^, and only those with negative local testing or in the unusual event of no testing for *EGFR* being available were included in SPECTA^4,11^.

Very few genomic alterations were identified in patients with pleural mesothelioma and thymic malignancies due to the limited number of patients included and the small size of the NGS panel run in SPECTAlung. For mesothelioma, we found the same top altered genes as in the TCGA cohort, such as loss of *CDKN2A* or *TP53*, *NF2* or *BAP1* mutations, none of which have currently been linked to a possible treatment. *CDKN2A* loss has been correlated with worst outcome^12^ or proposed as a diagnostic tool, as observed in more than 75% of mesothelioma^13^. *BAP1* is also frequently altered in mesothelioma, however a recent study highlights no difference in response to treatment (chemotherapy, immune checkpoint inhibitors or PARP inhibitors) between *BAP1* wild-type or mutant mesothelioma^14^. For thymic malignancies, we were unable to confirm findings from TCGA as *GTF2I* was not part of any of the panels used in SPECTAlung and the sample size was very small. Moreover, we were also unable to repeats the molecular profile of the Arcagen studies (n = 23) for which several mutations were found in the RAS/RAF pathway^15^, similarly to what was found in the TCGA cohort. This clearly highlight the need for further fundamental and clinical research for rare cancers and possibly NGS panels adaptations.

The SPECTAlung platform faced important operational challenges, the main one being the long turn-around time from patient registration to delivery of molecular results (above 200 days on average). Workflow adaptations were made to try improving the overall quality and TAT. Three successive central laboratories performed the molecular analysis throughout the project and some improvement was noticed regarding workflow uniformity and robustness with a decrease of TAT variability, once the latest sample workflow was established (SD of 104 for Oncomine vs 62.3 for Illumina). However, despite those adaptations, the slow TAT impaired the actionability of treatment recommendations and *in fine*, the treatment adaptations based on molecular tumor board recommendations. Even though treatment recommendations were suggested for 103 patients (19.5%), mostly for NSCLC, adaptation was limited to 15.5% of the recommendations. The very high attrition rate between number of patients eligible in the platform (n = 480) to treatment adaptation (n = 16, 3.3%) might be linked to the fact that half of SPECTAlung patients had early-stage disease and did not progress within the timeframe of the project, or died prior to receiving the results (for patients with advanced disease) due to the very long TAT. Another important aspect is limitation to treatment access when outside standard of care and clinical trials. Finally, it is important to note that the use of three different molecular tests hinder the comparability of the SPECTAlung patients and therefore the final analysis.

SPECTAlung was one of the first pan-European academic screening platform and here we highlighted the challenges and successes encountered. Developing a pan-European screening platform, albeit limited to thoracic malignancies, paved the way for a more global pan-tumor screening program, which could help with personalised treatment and new platform trials, by reducing costs, ensuring quality and reproducibility of analysis, and minimizing turn-around time.

The platform was set-up and functional in more than 7 countries and was useful, especially in alterations present in multiple tumour types with a low prevalence. However, the operational set-up and the TAT were long and hindered the relevance of testing for patient treatment. A centralised testing system with timely MTB needs to be more efficient than local labs, should deliver a broader spectrum of genes being tested, have more flexibility in choice of panel and panel content, including the use of liquid biopsy to decrease TAT and help with FFPE sample failure, and include option for the patients to be recruited into clinical trials. During the course of the project, we adapted the screening panel, to increase the number of sequenced genes. However, in SPECTAlung, we never used liquid biopsy, even if its role is now well-established for lung cancers. Moreover, novel biomarkers are emerging, especially linked with Antibody–drug Conjugates and new targeted therapy. Panel adaptation, but also use of new technologies and combination of other screening methods (such as IHC for protein expression) might be needed in the future.

Based on this experience and from other European platforms (MAPPYACTS (Gustave Roussy, France), Master and INFORM protocol (DKFZ, Germany)), the SPECTA platform has been adapted toward a translational research platform, to increase the scope and improve its global efficiency. Beyond providing molecular results to cancer patients, SPECTAlung is also a source of clinically annotated molecular data, close to real-world data, that could prove extremely valuable as control-arm for future clinical research on biomarker of rare incidence.

The area of personalized medicine and complex trials is developing actively. Even in the light of immunotherapy, biomarkers identifying best responders and rationale for potential combination therapies are being developed. Drugs are now being approved based on tumor agnostic molecular abnormalities, so that screening has to cover all indications, not only the drivers related to a specific tumor type. Therefore, the need for and harmonized screening access in Europe, especially for smaller hospitals or countries where the screening is not systemic is crucial to allow access to such therapies to all patients. This is especially true for non-small cell lung cancer, as a disease with a high number of potential actionable molecular alterations.

## Methods

Patients older than 18, with a diagnosis of any thoracic malignancy (including NSCLC, pleural mesothelioma and thymic malignancies) at any stage were enrolled in the program (NCT 02214134). All patients had provided written informed consent at the time of sample collection for genomic analysis. The SPECTAlung study was approved by several ethical committees (Ethische Commissie Onderzoek UZ/KU Leuven, in Belgium (S57513), Comité de protection des personnes "Ile-de-France VII", in France (15-030 (PP 15-001))).

### Sample workflow

At registration, a FFPE sample was sent to the central biobank (Gustave Roussy, France or IBBL, Luxembourg). A pathology review was carried out to assess sufficient quality for molecular analysis, based on tumour content (more than 100 viable tumour cells and more than 10% tumour content). Samples used in this project were processed and stored under the management of the Integrated Biobank of Luxembourg (IBBL) following ISO17025:2005 standards and the ISBER Best Practices.

### Workflows and screening platforms

Three screening platforms were used for SPECTAlung: 14 MG (spin off from the Sanger Institute, June to December 2015), Oncomine focus panel (ThermoFisher, December 2015 to August 2017) and Illumina TST-170 (September 2017 to November 2018). Molecular alterations were called using the 14MG proprietary pipeline for samples analysed by 14MG^1^. For samples analysed with Oncomine focus panel bioinformatics analysis were performed with Torrent Suite™ Server and Ion Reporter™ Server, which includes the Oncomine™ Variant Annotation Tool, according to Thermofisher standard recommendation. For samples analysed by Illumina, CNVs were called by the Illumina official pipeline but the EORTC pipeline was used for somatic variants and fusion calling. All methods were carried out in accordance with relevant guidelines and regulations. All genomic data generated for this study is available on EGA under study ID: EGAS00001004485 (https://ega-archive.org/studies/EGAS00001004485).

Genomic and clinical data can be shared upon request submitted to EORTC (Data sharing—EORTC : EORTC).

### Clinical database

All clinical information was collected centrally, in EORTC clinical database. For patient registration, complete information on patient demographics, medical history, and current disease situation was required. Follow-up visits were done according to local standards, and follow-up data were submitted to EORTC.

### Reporting and molecular tumour board

For all patients with molecular analysis results, a molecular report was shared with the treating-clinician and the case was discussed within an international virtual molecular tumour board (monthly), with local and expert clinicians, bioinformaticians and molecular biologists.

See supplementary material and methods for more information.

## Supplementary Information

Supplementary Information.

## Data Availability

All genomic data generated for this study is available on EGA under study ID: EGAS00001004485 (https://ega-archive.org/studies/EGAS00001004485). Genomic and clinical data can be shared upon request submitted to EORTC (Data sharing—EORTC : EORTC).
