# Supplementary material for: Results of screening in early and advanced thoracic malignancies in the EORTC pan-European SPECTAlung platform
Source: Sci Rep. 2022 May 18;12:8342. doi: 10.1038/s41598-022-12056-0 (PMC9117328; doi:10.1038/s41598-022-12056-0)
Supplement: Supplementary file 1 — Supplementary Information. [file 41598_2022_12056_MOESM1_ESM.docx]

**Supplementary material and methods**

*Additional data analysis*

FASTQ files were first aligned to hg19 reference genome using bwa-mem aligner v0.7.17 with default settings. Reads were further coordinate-sorted, indexed and lanes were merged using Samtools v1.9 [1]. Duplicate reads were identified using Picard MarkDuplicates [2]. BaseRecalibrator from GATK 4.0.10 toolkit [3] was used next for recalibration of scores with following options *–use-original-qualities –known-sites dbsnp milles indels_1000G,* as well as ApplyBQSR with these settings *--static-quantized-quals 10 --static-quantized-quals 20 --static-quantized-quals 30 --add-output-sam-program-record --use-original-qualities* [4]*.*

Somatic variants were called by Mutect2 in tumour-only mode and false positives are filtered out using MutectFilter with additional option *--max-germline-posterior 1* required to avoid germline risk as the variants were called in tumour-only mode [5].

Additional filtering was performed to remove all variants with coverage less than 250 and variant allele fraction (VAF) less than 0.05. Mutations were later annotated using pcgr tool version 0.7.0 with default settings for each tumour type [6].

Metrics were collected on every major step of the pipeline.

Fusions were called using both StarFusion version 1.7.0 and FusionCatcher version 1.10 with --skip-blat option [7,8]. Only fusions in genes covered by Illumina panel were considered for further analysis and annotated using scripts developed by EORTC. Tumour mutational burden (TMB) was calculated as a number of synonymous and non-synonymous single nucleotide variants (excluding variants with allele frequency greater than 0.01 in gnomAD and 1000G), divided by 0.524 Mb (size of genomic region covered in TST170 gene panel).

**Supplementary Figure 1**: OS follow-up time Kaplan Meier curve. OS follow-up times were estimated by the inverse Kaplan-Meier method (i.e. doing a curve with event in curve=censored data and censored in curve=event reached in the database). The median OS follow-up with 95% Confidence Intervals (calculated by Brookmeyer and Crowley technique [9]. Circles on the plot represent deaths.

| **Evaluable patients** | **Patients (N)** | **Observed Events (O)** | **Median (95% CI) (Years)** |
| --- | --- | --- | --- |
| Yes | 539 | 354 | 3.84 (3.59, 4.03) |

**Supplementary Table 1**: Genes covered by different panels used in SPECTAlung

| **Gene** | **TST170** | **Oncomine** | **14MG** |
| --- | --- | --- | --- |
| *ABL1* | Yes | Yes |  |
| *ACTG1* |  |  | Yes |
| *ACVR1B* |  |  | Yes |
| *ACVR2A* |  |  | Yes |
| *AGAP5* |  |  | Yes |
| *AJUBA* |  |  | Yes |
| *AKAP9* |  |  | Yes |
| *AKT1* | Yes | Yes | Yes |
| *AKT2* | Yes |  | Yes |
| *AKT3* | Yes | Yes | Yes |
| *ALK* | Yes | Yes | Yes |
| *ALKBH6* |  |  | Yes |
| *AMER1* |  |  | Yes |
| *APC* | Yes |  | Yes |
| *APH1A* |  |  | Yes |
| *AR* | Yes | Yes | Yes |
| *ARHGAP35* |  |  | Yes |
| *ARID1A* | Yes |  | Yes |
| *ARID1B* |  |  | Yes |
| *ARID2* |  |  | Yes |
| *ARID5B* |  |  | Yes |
| *ARNT* |  |  | Yes |
| *ASXL1* |  |  | Yes |
| *ASXL2* |  |  | Yes |
| *ATM* | Yes |  | Yes |
| *ATP1A1* |  |  | Yes |
| *ATP2B3* |  |  | Yes |
| *ATR* | Yes |  | Yes |
| *ATRX* |  |  | Yes |
| *AURKA* |  |  | Yes |
| *AXIN1* |  |  | Yes |
| *AXIN2* |  |  | Yes |
| *AXL* | Yes | Yes |  |
| *B2M* |  |  | Yes |
| *BAP1* | Yes |  | Yes |
| *BARD1* | Yes |  |  |
| *BCL2* | Yes |  |  |
| *BCL6* | Yes |  |  |
| *BCL9* |  |  | Yes |
| *BCLAF1* |  |  | Yes |
| *BCOR* |  |  | Yes |
| *BLM* |  |  | Yes |
| *BMPR2* |  |  | Yes |
| *BRAF* | Yes | Yes | Yes |
| *BRCA1* | Yes |  | Yes |
| *BRCA2* | Yes |  | Yes |
| *BRIP1* | Yes |  |  |
| *BTK* | Yes |  |  |
| *C11orf30* |  |  | Yes |
| *C2CD3* |  |  | Yes |
| *C3orf70* |  |  | Yes |
| *CACNA1D* |  |  | Yes |
| *CARD11* | Yes |  |  |
| *CASP1* |  |  | Yes |
| *CASP8* |  |  | Yes |
| *CBFB* |  |  | Yes |
| *CBWD1* |  |  | Yes |
| *CCDC120* |  |  | Yes |
| *CCDC6* |  |  | Yes |
| *CCND1* | Yes | Yes | Yes |
| *CCND2* | Yes |  | Yes |
| *CCND3* | Yes |  | Yes |
| *CCNE1* | Yes |  | Yes |
| *CD79A* | Yes |  |  |
| *CD79B* | Yes |  |  |
| *CDC73* |  |  | Yes |
| *CDH1* | Yes |  | Yes |
| *CDK12* | Yes |  | Yes |
| *CDK4* | Yes | Yes | Yes |
| *CDK6* | Yes | Yes | Yes |
| *CDK8* |  |  | Yes |
| *CDKN1A* |  |  | Yes |
| *CDKN1B* |  |  | Yes |
| *CDKN2A* | Yes |  | Yes |
| *CDKN2C* |  |  | Yes |
| *CEBPA* | Yes |  | Yes |
| *CEP290* |  |  | Yes |
| *CHD1* |  |  | Yes |
| *CHD1L* |  |  | Yes |
| *CHD3* |  |  | Yes |
| *CHD4* |  |  | Yes |
| *CHD5* |  |  | Yes |
| *CHD7* |  |  | Yes |
| *CHD8* |  |  | Yes |
| *CHEK1* | Yes |  |  |
| *CHEK2* | Yes |  | Yes |
| *CHMP3* |  |  | Yes |
| *chr14:59220408-59220378* |  |  | Yes |
| *chr22:29059272-29065303* |  |  | Yes |
| *chr3:123590727-123590709* |  |  | Yes |
| *chr6:13191015-13191013* |  |  | Yes |
| *chrX:11953208-11959433* |  |  | Yes |
| *CIC* |  |  | Yes |
| *CKS1B* |  |  | Yes |
| *CMTR2* |  |  | Yes |
| *CNBD1* |  |  | Yes |
| *CNOT1* |  |  | Yes |
| *CNOT3* |  |  | Yes |
| *COL2A1* |  |  | Yes |
| *COL5A1* |  |  | Yes |
| *CREBBP* | Yes |  | Yes |
| *CRKL* |  |  | Yes |
| *CSDE1* |  |  | Yes |
| *CSF1R* | Yes |  |  |
| *CSNK2A1* |  |  | Yes |
| *CTCF* |  |  | Yes |
| *CTNNA1* |  |  | Yes |
| *CTNNB1* | Yes | Yes | Yes |
| *CTNND2* |  |  | Yes |
| *CUL2* |  |  | Yes |
| *CUL3* |  |  | Yes |
| *CUL4B* |  |  | Yes |
| *CUX1* |  |  | Yes |
| *CYLD* |  |  | Yes |
| *DAXX* |  |  | Yes |
| *DCUN1D1* |  |  | Yes |
| *DDR2* | Yes | Yes | Yes |
| *DDX3X* |  |  | Yes |
| *DDX5* |  |  | Yes |
| *DICER1* |  |  | Yes |
| *DNMT3A* | Yes |  | Yes |
| *DRD5* |  |  | Yes |
| *E2F3* |  |  | Yes |
| *EEF1A1* |  |  | Yes |
| *EGFR* | Yes | Yes | Yes |
| *EIF1AX* |  |  | Yes |
| *EIF2AK3* |  |  | Yes |
| *EIF4A2* |  |  | Yes |
| *ELF3* |  |  | Yes |
| *ELK4* |  |  | Yes |
| *EML4* | Yes |  |  |
| *EP300* | Yes |  | Yes |
| *EPC1* |  |  | Yes |
| *ERBB2* | Yes | Yes | Yes |
| *ERBB3* | Yes | Yes | Yes |
| *ERBB4* | Yes | Yes | Yes |
| *ERC1* |  |  | Yes |
| *ERCC1* | Yes |  |  |
| *ERCC2* | Yes |  | Yes |
| *ERCC5* |  |  | Yes |
| *ERG* | Yes | Yes | Yes |
| *ESR1* | Yes | Yes | Yes |
| *ETS1* | Yes |  |  |
| *ETS2* |  |  | Yes |
| *ETV1* | Yes | Yes | Yes |
| *ETV4* | Yes | Yes | Yes |
| *ETV5* | Yes | Yes | Yes |
| *EWSR1* | Yes |  |  |
| *EZH1* |  |  | Yes |
| *EZH2* | Yes |  | Yes |
| *EZR* |  |  | Yes |
| *FADD* |  |  | Yes |
| *FAF1* |  |  | Yes |
| *FAM106A* |  |  | Yes |
| *FAM135B* |  |  | Yes |
| *FAM175A* | Yes |  |  |
| *FANCI* | Yes |  |  |
| *FANCL* | Yes |  |  |
| *FAS* |  |  | Yes |
| *FAT1* |  |  | Yes |
| *FBXW7* | Yes |  | Yes |
| *FGF1* | Yes |  |  |
| *FGF10* | Yes |  |  |
| *FGF14* | Yes |  |  |
| *FGF19* | Yes |  |  |
| *FGF2* | Yes |  |  |
| *FGF23* | Yes |  |  |
| *FGF3* | Yes |  |  |
| *FGF4* | Yes |  |  |
| *FGF5* | Yes |  |  |
| *FGF6* | Yes |  |  |
| *FGF7* | Yes |  |  |
| *FGF8* | Yes |  |  |
| *FGF9* | Yes |  |  |
| *FGFR1* | Yes | Yes | Yes |
| *FGFR2* | Yes | Yes | Yes |
| *FGFR3* | Yes | Yes | Yes |
| *FGFR4* | Yes | Yes | Yes |
| *FKBP9* |  |  | Yes |
| *FLI1* | Yes |  |  |
| *FLT1* | Yes |  |  |
| *FLT3* | Yes |  | Yes |
| *FOXA1* |  |  | Yes |
| *FOXA2* |  |  | Yes |
| *FOXL2* | Yes |  | Yes |
| *FOXO1* |  |  | Yes |
| *FOXP1* |  |  | Yes |
| *FOXP3* |  |  | Yes |
| *FRG1B* |  |  | Yes |
| *FRS2* |  |  | Yes |
| *FUBP1* |  |  | Yes |
| *G3BP2* |  |  | Yes |
| *GAB2* |  |  | Yes |
| *GATA3* |  |  | Yes |
| *GATA6* |  |  | Yes |
| *GEN1* | Yes |  |  |
| *GNA11* | Yes | Yes | Yes |
| *GNAQ* | Yes | Yes | Yes |
| *GNAS* | Yes |  | Yes |
| *GOPC* |  |  | Yes |
| *GPS2* |  |  | Yes |
| *GRB7* |  |  | Yes |
| *GRIN2A* |  |  | Yes |
| *GTPBP4* |  |  | Yes |
| *H3F3A* |  |  | Yes |
| *H3F3B* |  |  | Yes |
| *H3F3C* |  |  | Yes |
| *HDAC9* |  |  | Yes |
| *HIST1H2BD* |  |  | Yes |
| *HIST1H3B* |  |  | Yes |
| *HLA-A* |  |  | Yes |
| *HLA-B* |  |  | Yes |
| *HNF1A* | Yes |  | Yes |
| *HRAS* | Yes | Yes | Yes |
| *HSP90AB1* |  |  | Yes |
| *HSPA2* |  |  | Yes |
| *HSPA5* |  |  | Yes |
| *HSPA8* |  |  | Yes |
| *IDH1* | Yes | Yes | Yes |
| *IDH2* | Yes | Yes | Yes |
| *IGF2* |  |  | Yes |
| *IL6ST* |  |  | Yes |
| *IL7R* |  |  | Yes |
| *ING1* |  |  | Yes |
| *INPP4B* | Yes |  |  |
| *INTS4* |  |  | Yes |
| *IRF2* |  |  | Yes |
| *JAK1* |  | Yes |  |
| *JAK2* | Yes | Yes | Yes |
| *JAK3* | Yes | Yes |  |
| *JARID1B* |  |  | Yes |
| *JUN* |  |  | Yes |
| *KALRN* |  |  | Yes |
| *KAT6B* |  |  | Yes |
| *KCNJ5* |  |  | Yes |
| *KDM5C* |  |  | Yes |
| *KDM6A* |  |  | Yes |
| *KDR* | Yes |  | Yes |
| *KEAP1* |  |  | Yes |
| *KIAA1549* |  |  | Yes |
| *KIF5B* | Yes |  |  |
| *KIT* | Yes | Yes | Yes |
| *KLF4* |  |  | Yes |
| *KLF6* |  |  | Yes |
| *KLHL8* |  |  | Yes |
| *KMT2A* |  |  | Yes |
| *KMT2A (MLL)* | Yes |  |  |
| *KMT2C* |  |  | Yes |
| *KMT2D* |  |  | Yes |
| *KRAS* | Yes | Yes | Yes |
| *LAMP1* | Yes |  |  |
| *LARP4B* |  |  | Yes |
| *LCTL* |  |  | Yes |
| *LIFR* |  |  | Yes |
| *LMO1* |  |  | Yes |
| *LRRK2* |  |  | Yes |
| *LZTR1* |  |  | Yes |
| *MALAT1* |  |  | Yes |
| *MAP2K1* | Yes | Yes | Yes |
| *MAP2K2* | Yes | Yes | Yes |
| *MAP2K4* |  |  | Yes |
| *MAP3K1* |  |  | Yes |
| *MAP4K3* |  |  | Yes |
| *MAT2A* |  |  | Yes |
| *MAX* |  |  | Yes |
| *MBD2* |  |  | Yes |
| *MCL1* | Yes |  |  |
| *MDM2* | Yes |  | Yes |
| *MDM4* | Yes |  | Yes |
| *MECOM* |  |  | Yes |
| *MED12* |  |  | Yes |
| *MED17* |  |  | Yes |
| *MED29* |  |  | Yes |
| *MEF2C* |  |  | Yes |
| *MEN1* |  |  | Yes |
| *MET* | Yes | Yes | Yes |
| *MGA* |  |  | Yes |
| *MITF* |  |  | Yes |
| *MLH1* | Yes |  | Yes |
| *MLLT3* | Yes |  | Yes |
| *MLLT4* |  |  | Yes |
| *MORC4* |  |  | Yes |
| *MPL* | Yes |  |  |
| *MRE11A* | Yes |  |  |
| *MSH2* | Yes |  | Yes |
| *MSH3* | Yes |  |  |
| *MSH6* | Yes |  | Yes |
| *MTOR* | Yes | Yes | Yes |
| *MUTYH* | Yes |  |  |
| *MYB* |  |  | Yes |
| *MYC* | Yes | Yes | Yes |
| *MYCL1* | Yes |  | Yes |
| *MYCN* | Yes | Yes | Yes |
| *MYD88* | Yes |  |  |
| *NAP1L1* |  |  | Yes |
| *NAV3* |  |  | Yes |
| *NBN* | Yes |  |  |
| *NCOA2* |  |  | Yes |
| *NCOA3* |  |  | Yes |
| *NCOR1* |  |  | Yes |
| *NDUFC2* |  |  | Yes |
| *NF1* | Yes |  | Yes |
| *NF2* |  |  | Yes |
| *NFE2L2* |  |  | Yes |
| *NKX2-1* |  |  | Yes |
| *NLRP3* |  |  | Yes |
| *NOTCH1* | Yes |  | Yes |
| *NOTCH2* | Yes |  | Yes |
| *NOTCH3* | Yes |  | Yes |
| *NOTCH4* |  |  | Yes |
| *NPM1* | Yes |  | Yes |
| *NRAS* | Yes | Yes | Yes |
| *NRG1* | Yes |  |  |
| *NSD1* |  |  | Yes |
| *NTN4* |  |  | Yes |
| *NTRK1* | Yes | Yes |  |
| *NTRK2* | Yes | Yes | Yes |
| *NTRK3* | Yes | Yes |  |
| *NUP98* |  |  | Yes |
| *PALB2* | Yes |  |  |
| *PAX3* | Yes |  |  |
| *PAX5* |  |  | Yes |
| *PAX7* | Yes |  |  |
| *PBRM1* |  |  | Yes |
| *PCBP1* |  |  | Yes |
| *PDGFRA* | Yes | Yes | Yes |
| *PDGFRB* | Yes |  |  |
| *PGR* |  |  | Yes |
| *PHF6* |  |  | Yes |
| *PHLPP1* |  |  | Yes |
| *PHOX2B* |  |  | Yes |
| *PIAS3* |  |  | Yes |
| *PIK3CA* | Yes | Yes | Yes |
| *PIK3CB* | Yes |  | Yes |
| *PIK3CD* | Yes |  |  |
| *PIK3CG* | Yes |  |  |
| *PIK3R1* | Yes |  | Yes |
| *PIK3R2* |  |  | Yes |
| *PLCG1* |  |  | Yes |
| *PLEKHS1* |  |  | Yes |
| *PLXNB1* |  |  | Yes |
| *PMS2* | Yes |  |  |
| *POLE* |  |  | Yes |
| *POLQ* |  |  | Yes |
| *PPARG* | Yes | Yes |  |
| *PPFIA1* |  |  | Yes |
| *PPP2R1A* |  |  | Yes |
| *PPP2R2A* | Yes |  |  |
| *PPP6C* |  |  | Yes |
| *PREX2* |  |  | Yes |
| *PRKAB2* |  |  | Yes |
| *PRKAR1A* |  |  | Yes |
| *PRKCI* |  |  | Yes |
| *PRPF6* |  |  | Yes |
| *PTCH1* | Yes |  | Yes |
| *PTEN* | Yes |  | Yes |
| *PTGS1* |  |  | Yes |
| *PTPN11* | Yes |  | Yes |
| *PTPRB* |  |  | Yes |
| *QKI* |  |  | Yes |
| *RAC1* |  |  | Yes |
| *RAD21* |  |  | Yes |
| *RAD50* |  |  | Yes |
| *RAD51* | Yes |  |  |
| *RAD51AP1* |  |  | Yes |
| *RAD51B* | Yes |  |  |
| *RAD51C* | Yes |  |  |
| *RAD51D* | Yes |  |  |
| *RAD52* |  |  | Yes |
| *RAD54L* | Yes |  |  |
| *RAF1* | Yes | Yes | Yes |
| *RAP1B* |  |  | Yes |
| *RASA1* |  |  | Yes |
| *RASAL2* |  |  | Yes |
| *RB1* | Yes |  | Yes |
| *RBM10* |  |  | Yes |
| *RET* | Yes | Yes | Yes |
| *RHEB* |  |  | Yes |
| *RHOA* |  |  | Yes |
| *RICTOR* | Yes |  |  |
| *RIT1* |  |  | Yes |
| *RNF43* |  |  | Yes |
| *ROBO1* |  |  | Yes |
| *ROBO2* |  |  | Yes |
| *ROS1* | Yes | Yes | Yes |
| *RPL22* |  |  | Yes |
| *RPL5* |  |  | Yes |
| *RPS6KB1* | Yes |  |  |
| *RPSAP58* |  |  | Yes |
| *RSBN1L* |  |  | Yes |
| *RTEL1* |  |  | Yes |
| *RUNX1* |  |  | Yes |
| *RXRA* |  |  | Yes |
| *RYBP* |  |  | Yes |
| *SACS* |  |  | Yes |
| *SCAI* |  |  | Yes |
| *SCN11A* |  |  | Yes |
| *SERPINB13* |  |  | Yes |
| *SETBP1* |  |  | Yes |
| *SETD2* |  |  | Yes |
| *SETDB1* |  |  | Yes |
| *SF3B1* |  |  | Yes |
| *SHQ1* |  |  | Yes |
| *SKP2* |  |  | Yes |
| *SLC1A3* |  |  | Yes |
| *SLC26A3* |  |  | Yes |
| *SLIT2* |  |  | Yes |
| *SLX4* | Yes |  |  |
| *SMAD2* |  |  | Yes |
| *SMAD3* |  |  | Yes |
| *SMAD4* | Yes |  | Yes |
| *SMARCA4* |  |  | Yes |
| *SMARCB1* | Yes |  | Yes |
| *SMC1A* |  |  | Yes |
| *SMC3* |  |  | Yes |
| *SMG1* |  |  | Yes |
| *SMO* | Yes | Yes | Yes |
| *SMURF1* |  |  | Yes |
| *SOS1* |  |  | Yes |
| *SOX17* |  |  | Yes |
| *SOX2* |  |  | Yes |
| *SOX9* |  |  | Yes |
| *SPEN* |  |  | Yes |
| *SPOP* |  |  | Yes |
| *SRC* | Yes |  |  |
| *STAG2* |  |  | Yes |
| *STAG3* |  |  | Yes |
| *STAT5B* |  |  | Yes |
| *STK11* | Yes |  | Yes |
| *STK19* |  |  | Yes |
| *STX2* |  |  | Yes |
| *SUFU* |  |  | Yes |
| *SUZ12* |  |  | Yes |
| *SYNE1* |  |  | Yes |
| *TAF1* |  |  | Yes |
| *TBL1XR1* |  |  | Yes |
| *TBX3* |  |  | Yes |
| *TCEB1* |  |  | Yes |
| *TCF7L2* |  |  | Yes |
| *TCL1A* |  |  | Yes |
| *TCP11L2* |  |  | Yes |
| *TERT* | Yes |  | Yes |
| *TET2* | Yes |  | Yes |
| *TFDP1* |  |  | Yes |
| *TFRC* | Yes |  |  |
| *TGFBR2* |  |  | Yes |
| *THSD7B* |  |  | Yes |
| *TJP2* |  |  | Yes |
| *TMPRSS2* | Yes |  | Yes |
| *TNK2* |  |  | Yes |
| *TP53* | Yes |  | Yes |
| *TP53BP1* |  |  | Yes |
| *TP53TG3D* |  |  | Yes |
| *TPTE* |  |  | Yes |
| *TPX2* |  |  | Yes |
| *TRAF7* |  |  | Yes |
| *TRIM23* |  |  | Yes |
| *TRMT10C* |  |  | Yes |
| *TRRAP* |  |  | Yes |
| *TSC1* | Yes |  | Yes |
| *TSC2* | Yes |  | Yes |
| *TSHR* |  |  | Yes |
| *TXNDC8* |  |  | Yes |
| *TXNIP* |  |  | Yes |
| *U2AF1* |  |  | Yes |
| *VHL* | Yes |  | Yes |
| *WASF3* |  |  | Yes |
| *WDR74* |  |  | Yes |
| *WHSC1L1* |  |  | Yes |
| *WNK1* |  |  | Yes |
| *WT1* |  |  | Yes |
| *XRCC2* | Yes |  |  |
| *Yap1* |  |  | Yes |
| *YEATS4* |  |  | Yes |
| *ZFHX3* |  |  | Yes |
| *ZFP36L1* |  |  | Yes |
| *ZMYM2* |  |  | Yes |
| *ZMYM3* |  |  | Yes |
| *ZNF217* |  |  | Yes |
| *ZNF407* |  |  | Yes |
| *ZNF483* |  |  | Yes |
| *ZNF595* |  |  | Yes |
| *ZNF620* |  |  | Yes |
| *ZNF703* |  |  | Yes |
| *ZNF717* |  |  | Yes |
| *ZNF814* |  |  | Yes |
| *ZNRF3* |  |  | Yes |

**Supplementary Figure 2**: KRAS mutations and smoking in TCGA and MSKCC cohorts


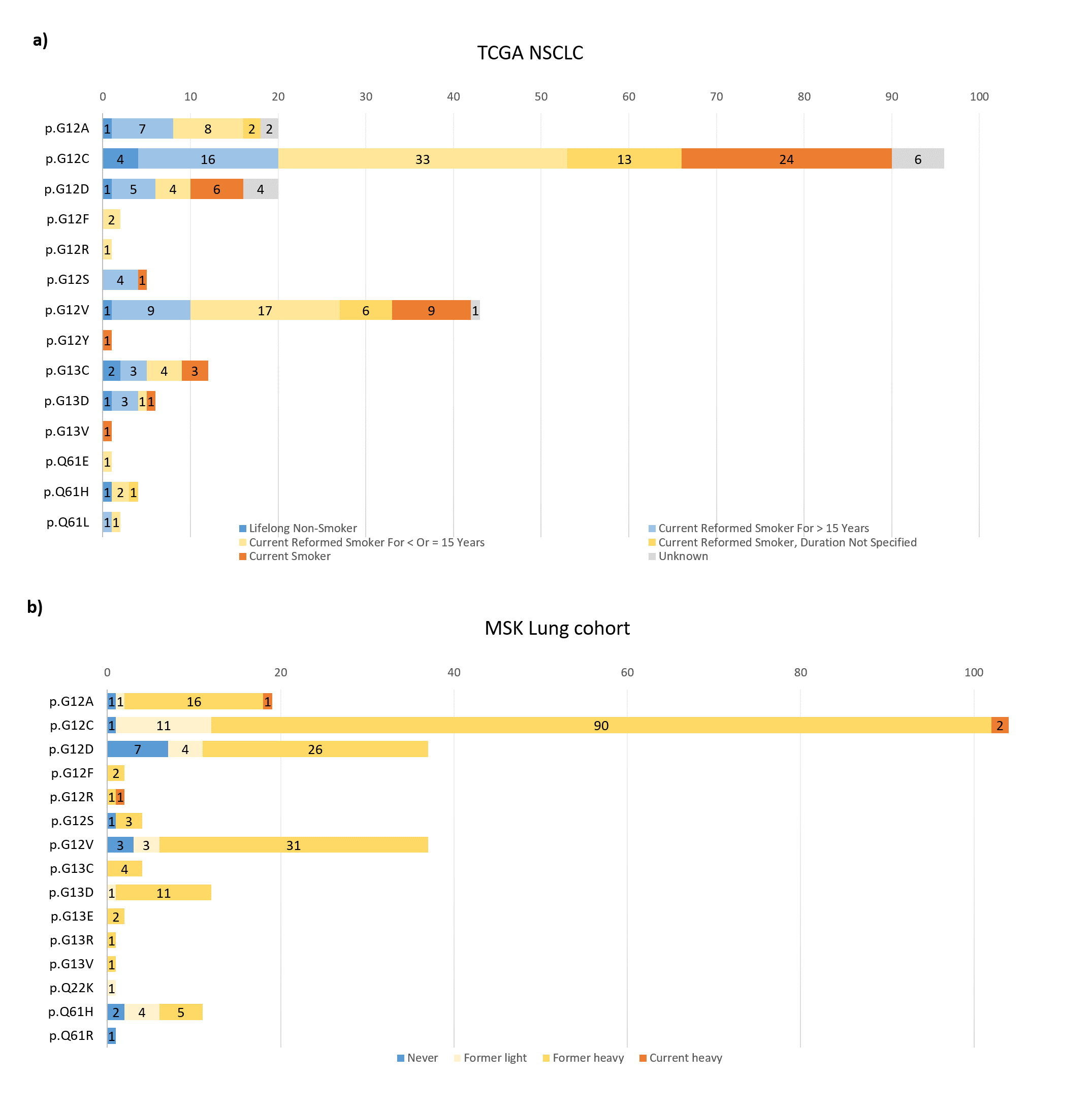


**References**

[1] Li H. [Heng Li - Compares BWA to other long read aligners like CUSHAW2] Aligning sequence reads, clone sequences and assembly contigs with BWA-MEM. ArXiv Prepr ArXiv 2013.

[2] Broad Institute. Picard toolkit. GitHub repository 2019 n.d. http://broadinstitute.github.io/picard/.

[3] Danecek P, Bonfield JK, Liddle J, Marshall J, Ohan V, Pollard MO, et al. Twelve years of SAMtools and BCFtools. Gigascience 2021. https://doi.org/10.1093/gigascience/giab008.

[4] McKenna A, Hanna M, Banks E, Sivachenko A, Cibulskis K, Kernytsky A, et al. The genome analysis toolkit: A MapReduce framework for analyzing next-generation DNA sequencing data. Genome Res 2010. https://doi.org/10.1101/gr.107524.110.

[5] Benjamin D, Sato T, Cibulskis K, Getz G, Stewart C, Lichtenstein L. Calling Somatic SNVs and Indels with Mutect2. BioRxiv 2019. https://doi.org/10.1101/861054.

[6] Nakken S, Fournous G, Vodák D, Aasheim LB, Myklebost O, Hovig E. Personal Cancer Genome Reporter: Variant interpretation report for precision oncology. Bioinformatics 2018. https://doi.org/10.1093/bioinformatics/btx817.

[7] Haas BJ, Dobin A, Li B, Stransky N, Pochet N, Regev A. Accuracy assessment of fusion transcript detection via read-mapping and de novo fusion transcript assembly-based methods. Genome Biol 2019. https://doi.org/10.1186/s13059-019-1842-9.

[8] Nicorici D, Satalan M, Edgren H, Kangaspeska S, Murumagi A, Kallioniemi O, et al. FusionCatcher - a tool for finding somatic fusion genes in paired-end RNA-sequencing data. 2014. https://doi.org/10.1101/011650.

[9] Brookmeyer R, Crowley J. A Confidence Interval for the Median Survival Time. Biometrics 1982. https://doi.org/10.2307/2530286.
